# High Neutrophil-to-Platelet Ratio Is Associated With Hemorrhagic Transformation in Patients With Acute Ischemic Stroke

**DOI:** 10.3389/fneur.2019.01310

**Published:** 2019-12-10

**Authors:** Weilei He, Yiting Ruan, Chengxiang Yuan, Qianqian Cheng, Haoran Cheng, Yaying Zeng, Yunbin Chen, Guiqian Huang, Huijun Chen, Jincai He

**Affiliations:** ^1^Department of Neurology, The First Affiliated Hospital of Wenzhou Medical University, Wenzhou, China; ^2^Department of Mental Health, Mental Health School, Wenzhou Medical University, Wenzhou, China

**Keywords:** neutrophil-to-platelet ratio, stroke, hemorrhagic transformation, parenchymal hematoma, outcome

## Abstract

**Background:** Hemorrhagic transformation (HT) is a complication that may cause neurological deterioration in patients with acute ischemic stroke. Both neutrophil and platelet have been associated with the stroke progression. The aim of this study was to explore the relationship between neutrophil-to-platelet ratio (NPR) and HT after acute ischemic stroke.

**Methods:** A total of 279 stroke patients with HT were consecutively recruited. HT was diagnosed using magnetic resonance imaging (MRI) or computed tomography (CT) and classified into hemorrhagic infarction (HI) and parenchymal hematoma (PH). Blood samples for neutrophil and platelet counts were obtained at admission. Meanwhile, 270 age- and gender-matched controls without HT were included for comparison.

**Results:** Among the patients with HT, 131 patients had PH and 148 patients had HI. NPR was higher in patients with PH than those with HI or non-HT [36.8 (23.7–49.2) vs. 26.6 (17.9–38.3) vs. 19.1 (14.8–24.8), *P* < 0.001]. After adjustment for potential confounders, high NPR remained independently associated with the increased risk of HT (OR = 2.000, 95% CI: 1.041–3.843, *P* = 0.037). NPR (>39.9) was independently associated with PH (OR = 2.641, 95% CI: 1.308–5.342, *P* = 0.007).

**Conclusions:** High NPR was associated with the increased risk of HT especially PH in patients with acute ischemic stroke.

## Introduction

Acute ischemic stroke is among the leading causes of mortality and long-term morbidity throughout the world (1). Hemorrhagic transformation (HT) is a common and serious complication after acute ischemic stroke (2). Patients with HT were vulnerable to experience neurological deterioration, worse functional outcome and increased mortality (3). Moreover, HT is categorized into parenchymal hematoma (PH) and hemorrhagic infarction (HI) based on the radiological appearance (4). A large population-based prospective study revealed that PH rather than HI was an independent risk factor for death or disability (5). Therefore, early recognition of HT especially PH is essential for the appropriate management and better prognosis in patients with acute ischemic stroke.

Neutrophil, the key role in the innate immune response, has been found to be associated with ischemic stroke and HT (6, 7). One study comparing the prognostic value of different inflammatory factors indicated that neutrophil was a more sensitive indicator for cardiovascular mortality compared with other subsets of leukocyte and C-reactive protein (8). The activation of neutrophil after stroke could contribute to large infarct volume, blood-brain barrier (BBB) disruption and HT (9). Higher counts of neutrophil after ischemic stroke were associated with symptomatic intracranial hemorrhage and worse functional outcome (10). Moreover, the reduction in brain neutrophil recruitment through the inhibition of nod-like receptor protein 3 (NLRP3) could preserve the integrity of BBB and attenuate HT (11).

Platelet has been considered as a necessary factor against HT following ischemia/reperfusion because of its hemostatic function (12). The decrease in platelet count after ischemic stroke, caused by hemodilution of excessive fluid replacement (13), was correlated with the high rates of HT and even symptomatic HT (14, 15). Except for its hemostatic function, platelet also plays an important role in the inflammatory response (16). The interaction between neutrophil and platelet was involved in the process of vascular injury after ischemic stroke (16). Platelet could facilitate neutrophil recruitment and extravasation into brain parenchyma after stroke, and the amount of infiltrated neutrophil was correlated with stroke progression (17, 18). Moreover, high neutrophil-platelet complex formation may increase the risk of stroke in patients with symptomatic carotid stenosis (19). Several studies found that the activation and aggregation of neutrophil and platelet within cerebral microvessels resulted in vascular inflammation and BBB dysfunction following ischemic-reperfusion injury (20, 21).

Increased neutrophil count and decreased platelet count may be associated with poor functional outcome in patients with HT (22, 23). A recent study found that high neutrophil-to-platelet ratio (NPR) was associated with long-term poor outcome in patients with acute ischemic stroke (24). Indeed, NPR shows its advantage in revealing information about the crosstalk between inflammation and hemostasis, and has been suggested as a useful and rapid screening tool to assess systemic inflammation in infective endocarditis (25). However, to date, no study has investigated the relationship between NPR and HT after ischemic stroke. The present study was designed to explore whether high NPR was associated with HT especially PH in patients with acute ischemic stroke.

## Materials and Methods

### Subjects

This was a retrospective study of HT patients who had been consecutively admitted to the Stroke Unit at the First Affiliated Hospital of Wenzhou Medical University between October 2011 and September 2018. The exclusion criteria were as follows: (1) acute infection within 2 weeks before admission or chronic infection; and (2) cancer, severe hepatic or renal diseases. Meanwhile, 270 age- and gender-matched controls without HT were included for comparison.

The study was approved by the ethics committee of the First Affiliated Hospital of Wenzhou Medical University, and the protocol followed the local ethics criteria for human research. Although written informed consent was not obtained for this study because of the retrospective design, it was obtained for data collection from our stroke registry.

### Clinical and Radiological Variables

Data were collected containing demographics (age and gender), risk factors (hypertension, diabetes mellitus, coronary heart disease, atrial fibrillation, history of stroke, and cigarette smoking), systolic blood pressure, diastolic blood pressure, the Trial of ORG10172 in the Acute Stroke Treatment (TOAST) classification and treatment in hospital (thrombolysis, antiplatelet and anticoagulation). The stroke severity was assessed by the National Institutes of Health Stroke Scale (NIHSS) at admission (26). Functional outcome was assessed by the Barthel Index (BI) at discharge, and the BI <60 was defined as poor functional outcome (27).

All patients underwent brain Computed Tomography (CT)/Magnetic Resonance Imaging (MRI) scans at admission, at day 4 (±2) and at any clinical worsening. HT was diagnosed using follow-up CT/MRI scans, and classified into PH and HI according to the recommendations of European Cooperative Acute Stroke Study (ECASS) II classification (28). PH was defined as hemorrhage with a mass effect. HT was determined separately by two neurologists blinded to clinical data, and the third was consulted when a divergence occurred. Moreover, the infarct location was divided into two groups: anterior circulation included frontal, parietal, lateral temporal cortical and subcortical regions, internal capsule, and basal ganglia; posterior circulation included brainstem, cerebellum, thalamus, medial temporal, and occipital regions (29). The infarct size was categorized as follows: less than one-half of a lobe was classified as small infarct volume, and more than one-half of a lobe was classified as large infarct volume (30).

### Laboratory Test

Blood samples were collected at admission in the Department of Emergency of our hospital and were obtained from the antecubital vein. The counts of leukocyte, neutrophil, lymphocyte, monocyte and platelet were obtained. The NPR was calculated by neutrophil count (×10^9^L) ×1000/platelet count (×10^9^/L). NPR was further divided into tertiles (tertile 1, tertile 2, tertile 3) in all patients and HT patients, respectively. The leukocyte-platelet ratio was calculated by leukocyte count (×10^9^L) ×1000/platelet count (×10^9^/L). The lymphocyte-platelet ratio was calculated by lymphocyte count (×10^9^L) ×1000/platelet count (×10^9^/L). The monocyte-platelet ratio was calculated by monocyte count (×10^9^L) ×1000/platelet count (×10^9^/L).

### Statistical Analysis

All patients were divided into HT and non-HT, and the HT group was further divided into PH and HI. The data were displayed as mean (standard deviation, SD) or median (interquartile range, IQR) for the continuous variables and percentages for the categorical variables. Student's *t*-test, analysis of variance (ANOVA) or Mann–Whitney U-test were applied for continuous variables, while the Chi-squared test was applied for proportions. On the one hand, receiver-operating characteristic (ROC) curves analysis were used to determine diagnostic accuracy of HT, and the cut-off values of NPR were calculated according to the Youden index. The area under the ROC curve (AUC) was considered as a critical diagnostic index. One the other hand, the levels of NPR were analyzed according to the degree of HT, while the degree of HT was also compared according to the NPR tertiles. Furthermore, multiple logistic regression analysis was used to evaluate whether NPR was associated with the incidence of HT and PH. For HT, model 1 was adjusted for age and sex; model 2 was adjusted for the variables in model 1 plus the factors that had already been established as predictors of HT (diabetes mellitus, atrial fibrillation, systolic blood pressure, large infarct volume, baseline NIHSS, anticoagulant and thrombolysis); and model 3 was adjusted for all the variables in model 2 plus the factors that significantly differed between the HT groups on the univariate analysis (coronary heart disease, anterior circulation and antiplatelet). For PH, model 1 was adjusted for age and sex; and model 2 was adjusted for the variables in model 1 plus the factors that had already been established as predictors of HT and that significantly differed between the outcome groups on the univariate analysis (diabetes mellitus, atrial fibrillation, systolic blood pressure, large infarct volume, baseline NIHSS, anticoagulant and thrombolysis). Odds ratios (ORs) and 95% confidence intervals (CIs) were calculated. *P* < 0.05 at two-tailed was considered statistically significant. All statistical analyses were performed on SPSS for Windows, version 23.0 (SPSS Inc., Chicago, IL, USA).

## Results

### Characteristics of Patients With HT/PH

This study enrolled 549 patients: 375 men (68.3%) and 174 women (31.7%). Their mean age was 69.0 ± 12.3 years. Among the 279 patients with HT, 131 patients had PH and 148 patients had HI. Clinical characteristics in patients with HT/PH and those without are summarized in Table 1. Patients with HT/PH were more likely to have atrial fibrillation, high baseline NIHSS and large infarct volume. The therapy of thrombolysis, anticoagulant and antiplatelet were more frequently observed in patients with HT than those without HT. Patients with PH had higher proportion of poor functional outcome compared with those with HI.

**Table 1:** Differences of the characteristics according to the subcategorized groups of HT.

	**All patients (*n =* 549)**			**Patients with HT (*n =* 279)**		
	**No HT (*n =* 270)**	**HT (*n =* 279)**	***P-*value**	**HI (*n =* 148)**	**PH (*n =* 131)**	***P-*value**
Demographics						
Age (y), mean ± SD	69.1 ± 12.1	68.9 ± 12.6	0.815	69.3 ± 11.8	68.4 ± 13.4	0.548
Male, *n* (%)	181 (67.0)	85 (30.5)	0.530	103 (69.6)	91 (69.5)	0.981
Risk factors, *n* (%)						
Hypertension	189 (70.0)	177 (63.4)	0.103	99 (66.9)	78 (59.5)	0.203
Diabetes mellitus	75 (27.8)	69 (24.7)	0.417	35 (23.6)	34 (26.0)	0.656
Coronary heart disease	14 (5.2)	32 (11.5)	0.008^*^	18 (12.2)	14 (10.7)	0.700
Atrial fibrillation	26 (9.7)	104 (37.3)	<0.001^**^	43 (29.1)	61 (46.6)	0.003^*^
History of stroke	31 (11.5)	40 (14.3)	0.319	25 (16.9)	15 (11.5)	0.196
Cigarette smoking	115 (42.6)	122 (43.7)	0.788	68 (45.9)	54 (41.2)	0.427
SBP (mmHg), mean ± SD	158 ± 23	149 ± 22	<0.001^**^	152 ± 22	146 ± 22	0.021^*^
DBP (mmHg), mean ± SD	82 ± 14	83 ± 14	0.546	83 ± 14	82 ± 14	0.505
Baseline NIHSS, median (IQR)	3 (1–5)	10 (5–13)	<0.001^**^	7 (3–12)	11 (7–14)	<0.001^**^
Stroke mechanisms, *n* (%)			<0.001^**^			0.002^*^
Large-artery atherosclerosis	245 (90.7)	157 (56.3)		97 (65.5)	60 (45.8)	
Cardioembolism	20 (7.4)	118 (42.3)		49 (33.1)	69 (52.7)	
Others	5 (1.9)	4 (1.4)		2 (1.4)	2 (1.5)	
Infarct location, *n* (%)						
Anterior circulation	183 (67.8)	257 (92.1)	<0.001^**^	133 (89.9)	124 (94.7)	0.138
Posterior circulation	126 (46.7)	117 (41.9)	0.265	68 (45.9)	49 (37.4)	0.149
Large infarct volume, n (%)	5 (1.9)	117 (41.9)	<0.001^**^	51 (34.5)	66 (50.4)	0.007^*^
Treatment in hospital, *n* (%)						
Thrombolysis	5 (1.9)	20 (7.2)	0.003^*^	7 (4.7)	13 (9.9)	0.093
Anticoagulant	26 (9.7)	84 (30.1)	<0.001^**^	38 (25.7)	46 (35.1)	0.086
Antiplatelet	248 (92.9)	154 (55.2)	<0.001^**^	86 (58.1)	68 (51.9)	0.299
Neutrophil count, mean ± SD	4.2 ± 1.4	6.3 ± 3.3	<0.001^**^	5.7 ± 3.0	7.0 ± 3.6	0.001
Platelet count, mean ± SD	207.0 ± 51.2	198.5 ± 67.3	0.096	203.5 ± 66.7	190.0 ± 63.4	0.085
NPR, median (IQR)	19.1 (14.8–24.8)	30.1 (20.5–44.7)	<0.001^**^	26.6 (17.9–38.3)	36.8 (23.7–49.2)	<0.001^**^

*HT, hemorrhagic transformation; HI, hemorrhagic infarct; PH, parenchymal hematoma; SD, standard deviation; SBP, systolic blood pressure; DBP, diastolic blood pressure; IQR, interquartile range; NPR, neutrophil-to-platelet ratio*;

* *P < 0.05*;

** *P < 0.001*.

Neutrophil count was significantly higher in the patients with PH than those with HI or non-HT [7.0 ± 3.6 vs. 5.7 ± 3.0 vs. 4.2 ± 1.5, *P* < 0.001]. Platelet count was significantly lower in the patients with PH than those with HI or non-HT [190.3 ± 63.2 vs. 205.8 ± 70.2 vs. 207.0 ± 51.2, *P* = 0.025]. According to the ROC curves, with an AUC of 0.733, NPR showed a greater discriminatory ability compared with neutrophil count [AUC 0.727, 95% CI (0.685–0.769), *P* < 0.001] and platelet count [AUC 0.570, 95% CI (0.521–0.617), *P* = 0.005] (Supplementary Figure 1). With an AUC of 0.733, NPR also showed a greater discriminatory ability compared with leukocyte-platelet ratio [AUC 0.695, 95% CI (0.651–0.740), *P* < 0.001], lymphocyte-platelet ratio [AUC 0.355, 95% CI (0.308–0.401), *P* < 0.001], and monocyte-platelet ratio [AUC 0.664, 95% CI (0.618–0.710), *P* < 0.001] (Supplementary Figure 2).

### Association Between NPR and HT/PH

**Figure 1:**
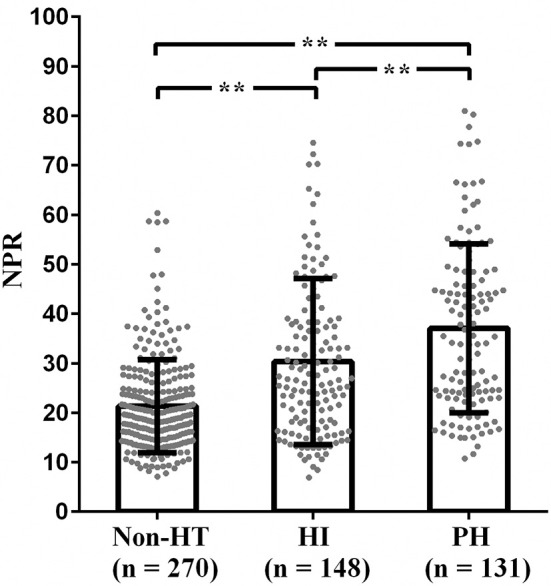
The levels of NPR in the subcategorized groups of HT. (1) The NPR was higher in patients with HI than those with non-HT [26.6 (17.9–38.3) vs. 19.1 (14.8–24.8), *P* < 0.001]; (2) The NPR was higher in patients with PH than those with non-HT [36.8 (23.7–49.2) vs. 19.1 (14.8–24.8), *P* < 0.001]; (3) The NPR was higher in patients with PH than those with HI [36.8 (23.7–49.2) vs. 26.6 (17.9–38.3)], *P* < 0.001). NPR, neutrophil-to-platelet ratio; HT, hemorrhagic transformation; HI, hemorrhagic infarct; PH, parenchymal hematoma; **P < 0.001.

**Figure 2:**
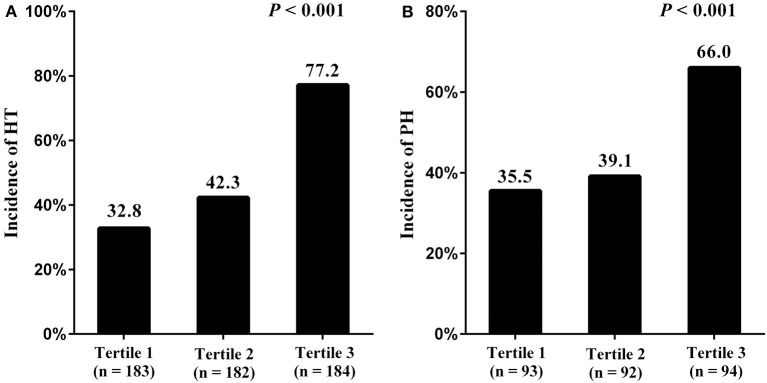
Incidence of HT and PH stratified by NPR tertiles [NPR tertiles in all patients: tertile 1, <18.4; tertile 2, 18.4–29.1; tertile 3, >29.1 **(A)**. NPR tertiles in patients with HT: tertile 1, <23.9; tertile 2, 23.9–39.9; tertile 3, >39.9 **(B)**]. NPR, neutrophil-to-platelet ratio; HT, hemorrhagic transformation; PH, parenchymal hematoma.

**Figure 3:**
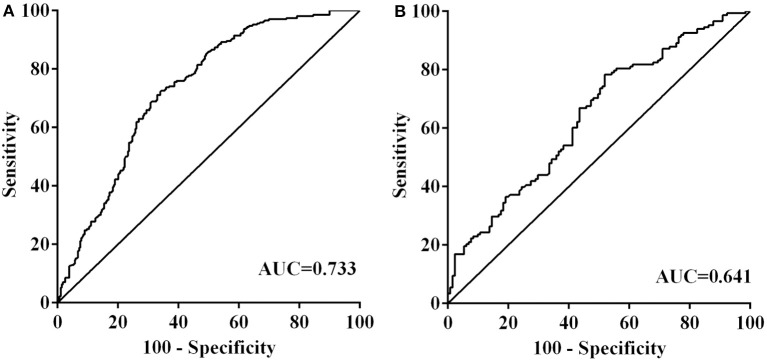
Receiver operator characteristic analysis of neutrophil-to-platelet ratio for predicting hemorrhagic transformation **(A)** and parenchymal hematoma **(B)**. AUC, area under the curve.

NPR was significantly higher in the patients with PH than those with HI or non-HT [36.8 (23.7–49.2) vs. 26.6 (17.9–38.3) vs. 19.1 (14.8–24.8), *P* < 0.001] (Figure 1). Furthermore, all patients were divided into three subgroups according to tertiles of NPR levels (tertile 1, < 18.4; tertile 2, 18.4–29.1; and tertile 3, >29.1). Patients with high NPR had a higher incidence of HT compared with those with middle or low NPR, respectively (77.2% vs. 42.3% vs. 32.8%; *P* < 0.001) (Figure 2A). With all patients taken as a whole, the incidence of HT taken as a dependent variable and low NPR taken as the reference used for NPR in the logistic analysis. Highest NPR was associated with the incidence of HT after adjustment for variables with clinical significance (model 2: OR = 2.503, 95% CI: 1.381–4.537, *P* = 0.002). The association remained significant after adjusting for factors that significantly differed between the HT groups on the univariate analysis (model 3: OR = 2.000, 95% CI: 1.041–3.843, *P* = 0.037). Atrial fibrillation, large infarct volume, baseline NIHSS and anterior circulation were also risk factors of HT (Table 2). According to the ROC curves, the optimal cut-off value for NPR as a diagnostic marker of HT was 24.24, with a sensitivity of 65.9% and a specificity of 72.6% [AUC 0.733, 95% CI (0.691–0.775), *P* < 0.001] (Figure 3). The NPR above the cut-off (24.24) remained independently associated with HT after adjustment for confounding variables (OR 2.069, 95% CI 1.195–3.584, *P* = 0.009).

**Table 2:** Factors associated with HT by multivariate logistic regression analysis.

	**Model 1**		**Model 2**		**Model 3**	
	**OR (95% CI)**	***P*-value**	**OR (95% CI)**	***P*-value**	**OR (95% CI)**	***P*-value**
**NPR**						
Tertile 1	Reference		Reference		Reference	
Tertile 2	1.521 (0.991–2.335)	0.055	0.946 (1.299–1.812)	0.837	1.017 (0.575–1.800)	0.953
Tertile 3	7.245 (4.525–11.600)	<0.001	7.839 (2.842–4.301)	0.005	2.000 (1.041–3.843)	0.037
Atrial fibrillation			2.627 (1.353–5.102)	0.004	2.514 (1.242–5.089)	0.010
Large infarct volume			7.839 (2.842–21.625)	<0.001	4.810 (1.679–13.780)	0.003
Baseline NIHSS			1.249 (1.175–1.329)	<0.001	1.256 (1.176–1.343)	<0.001
Anterior circulation					4.133 (2.068–8.261)	<0.001
Antiplatelet					0.213 (0.109–0.417)	<0.001

*Model 1 was adjusted for age and gender. Model 2 was adjusted for the variables in model 1 plus the factors that had already been established as predictors of HT, including diabetes mellitus, atrial fibrillation, systolic blood pressure, large infarct volume, baseline NIHSS, anticoagulant and thrombolysis. Model 3 was adjusted for all the variables in model 2 plus the factors that significantly differed between the HT groups on the univariate analysis, including coronary heart disease, anterior circulation and antiplatelet. NPR, neutrophil-to-platelet ratio; HT, hemorrhagic transformation; OR, odd ratio; CI, confidence interval; NIHSS, National Institutes of Health Stroke Scale*.

When the HT patients were classified into tertiles of NPR levels (tertile 1, <23.9; tertile 2, 23.9–39.9; and tertile 3, >39.9), HT patients with high NPR had a higher incidence of PH compared with those with middle or low NPR, respectively (66.0% vs. 39.1% vs. 35.5%; *P* < 0.001) (Figure 2B). With HT patients taken as a whole, the incidence of PH taken as a dependent variable and low NPR taken as the reference used for NPR in the logistic analysis. Highest NPR remained independently associated with the incidence of PH (model 2: OR = 2.641, 95% CI: 1.308–5.342, *P* = 0.007), after adjustment for confounding factors. Baseline NIHSS was also a risk factor of PH (Table 3). According to the ROC curve, the optimal cut-off value for NPR as a diagnostic marker of PH was 39.40, with a sensitivity of 48.1% and a specificity of 78.4% [AUC 0.641, 95% CI (0.577–0.706), *P* < 0.001] (Figure 3). The NPR above the cut-off (39.40) remained independently associated with PH after adjustment for confounding variables (OR 2.724, 95% CI 1.497–4.959, *P* = 0.001). Moreover, similar conclusion was attained when the NPR level was continuous variable in the logistic regression analysis [HT (OR = 1.033 95% CI, 1.009–1.057, *P* = 0.006) and PH (OR = 1.020, 95% CI, 1.004–1.036, *P* = 0.013)].

**Table 3:** Factors associated with PH by multivariate logistic regression analysis.

	**Model 1**		**Model 2**	
	**OR (95%CI)**	***P*-value**	**OR (95%CI)**	***P-*value**
**NPR**				
Tertile 1	Reference		Reference	
Tertile 2	1.249 (0.680–2.293)	0.474	0.975 (0.512–1.855)	0.938
Tertile 3	3.971 (2.121–7.437)	<0.001	2.641 (1.308–5.342)	0.007
Atrial fibrillation			1.492 (0.844–2.638)	0.169
Large infarct volume			1.073 (0.607–1.899)	0.808
Baseline NIHSS			1.074 (1.016–1.135)	0.011

*Model 1 was adjusted for age and gender. Model 2 was adjusted for the variables in model 1 plus the factors that had already been established as predictors of HT and that significantly differed between the outcome groups on the univariate analysis, including diabetes mellitus, atrial fibrillation, systolic blood pressure, large infarct volume, baseline NIHSS, anticoagulant and thrombolysis. NPR, neutrophil-to-platelet ratio; PH, parenchymal hematoma; OR, odd ratio; CI, confidence interval; NIHSS, National Institutes of Health Stroke Scale*.

## Discussion

To the best of our knowledge, this is the first study to explore the association between NPR and HT in patients with acute ischemic stroke. The present study indicated that high NPR was associated with an increased risk of HT after acute ischemic stroke. Moreover, we also found that HT patients with higher NPR were more likely to develop PH.

Accumulating studies have indicated that HT was correlated with ischemia/reperfusion injury, which was mainly attributed to BBB disruption, hemostatic dysfunction, oxidative stress and inflammation (31). One study investigating the relationship between BBB permeability and degree of HT found that higher BBB permeability may contribute to major intracranial bleeding in patients with acute ischemic stroke (32). Another neuroimaging study found that mild BBB disruption in ischemic brain was reversible due to the early reperfusion, while severe BBB disruption after sustained ischemia may increase the risk of HT and PH (33). Moreover, the increase in matrix metalloproteinase-9 (MMP-9) during the inflammatory phase of ischemic stroke was related to HT, and a 24 h peak of MMP-9 occurred before PH (34). The inhibition of MMP-9 expression with baicalin could maintain the BBB integrity, attenuate thrombolysis-induced HT and improve the prognosis in patients with acute ischemic stroke (35).

Several studies have indicated that neutrophil played an important role in the BBB disruption after stroke (22, 36). Neutrophils could release pro-inflammatory factors, reactive oxygen species and proteolytic enzymes, leading to BBB disruption and brain injury (9, 37). Previous studies found that neutrophil was the major source of MMP-9 acting on the BBB, which may result in symptomatic HT and poor outcome after ischemic stroke (10, 38). Higher neutrophil count was correlated with higher intracerebral hemorrhage volume at admission (39). The suppression of neutrophil recruitment was found to reduce hemorrhage volume and attenuate the severity of HT (11). While one study showed that low neutrophil count was associated with an increased risk of hematoma expansion during the hyperacute phase of intracerebral hemorrhage (40). One explanation for the contradictory findings may be that the effect of neutrophil on the vascular injury may be mediated by platelet, and the role of neutrophil-platelet interaction in the vascular inflammation may be varied during the different phase of intracerebral hemorrhage (41). The interaction between neutrophil and platelet could enhance the reactive oxygen species generation and exasperate the vascular injury (42), despite of the procoagulant properties of the activated neutrophils (43, 44). Moreover, platelet serves as a major contributor of several pro-inflammatory factors like tumor necrosis factor-α (41), which contributed to the increase in activated neutrophils and neutrophil-platelet aggregates (45). An animal study found that the depletion of platelet reduced the neutrophil recruitment and vascular inflammation after the occlusion of the middle cerebral artery (46).

In addition, a review of neutrophil-platelet interactions revealed that activated platelets were involved in the release of inflammatory mediators, neutrophil accumulation and the increase in vascular permeability (16). Platelet-endothelial interactions might prevent or heal neutrophil-induced vascular injury through the local release of soluble vasculoprotective factors (47). Hemostatic function of platelets was also attributed to the formation of plugs and clotting at the site of vessel injury (48), which helped to maintain the BBB integrity (49). Systemic inflammation was often accompanied by low platelet count, and consumption of platelet may be caused by the immunological process in the circulation (41). Therefore, it can be inferred that patients with higher NPR may have more severe disruption of BBB, leading to higher incidence of HT and PH. The better comprehension of neutrophil-platelet interactions may pave the way to promote neuroprotection and vascular repair in response to systematic inflammation after ischemic stroke (16).

In the present study, patients with PH had greater stroke severity and worse functional outcome compared with those with HI. A recent study supporting our findings showed that PH rather than HI was independently associated with poor functional outcome after thrombectomy in patients with acute large vessel occlusion (50). Some studies even indicated that patients with HI had a better functional outcome than those without HT because HI was a sign of early revascularization and better reperfusion (51, 52). Consistent with previous studies, we found that atrial fibrillation and large infarct volume were risk factors of HT (2). Moreover, we also found that anterior circulation was associated with HT. One possible reason for this phenomenon may be that patients with anterior circulation stroke were more likely to have greater stroke severity, atrial fibrillation and vessel occlusion, which were correlated with HT (2, 53).

There are some limitations in this study. First, the NPR was recorded only once, and it is necessary to investigate the association of dynamic changes in NPR after stroke with HT. Second, we did not explore the mechanisms underlying the influence of neutrophil and platelet on the BBB disruption in animal models, which should be conducted in our future work. Third, the functional outcome in stroke patients was assessed by BI, and our prospective studies will be conducted to better assess the functional outcome by modified Rankin scale (mRS). Fourth, the infarct size was taken as categorical variables in the analysis, and it is better to estimate the infarct size using the Alberta Stroke Program Early CT Score (ASPECTS) system by trained radiologists. Finally, considering the role of inflammation in the HT is still complex, more studies should be taken to further and better record more inflammatory index in the future.

## Conclusions

The present study demonstrated that high NPR was associated with the risk of HT and PH in patients with acute ischemic stroke. These findings may help clinicians to identify which stroke patients are at high risk of HT especially PH, and thus conduct appropriate therapy and CT scan in this population. Considering the limitations in the present study, this relationship needs further investigations in future.

## Data Availability Statement

The raw data supporting the conclusions of this manuscript will be made available by the corresponding author, without undue reservation, to any qualified researcher upon reasonable request.

## Ethics Statement

The studies involving human participants were reviewed and approved by The First Affiliated Hospital of Wenzhou Medical University. Written informed consent for participation was not required for this study in accordance with the national legislation and the institutional requirements.

## Author Contributions

WH and JH designed the project. WH did the statistical analyses and wrote the manuscript draft. WH, YR, CY, QC, HCheng, YZ, YC, GH, and HChen screened and extracted data. All authors have made an intellectual contribution to the manuscript and approved the submission.

### Conflict of Interest

The authors declare that the research was conducted in the absence of any commercial or financial relationships that could be construed as a potential conflict of interest.
