# Supplementary material for: High Neutrophil-to-Platelet Ratio Is Associated With Hemorrhagic Transformation in Patients With Acute Ischemic Stroke
Source: Front Neurol. 2019 Dec 10;10:1310. doi: 10.3389/fneur.2019.01310 (PMC6914868; doi:10.3389/fneur.2019.01310)
Supplement: Supplementary file 2 [file Data_Sheet_2.PDF]

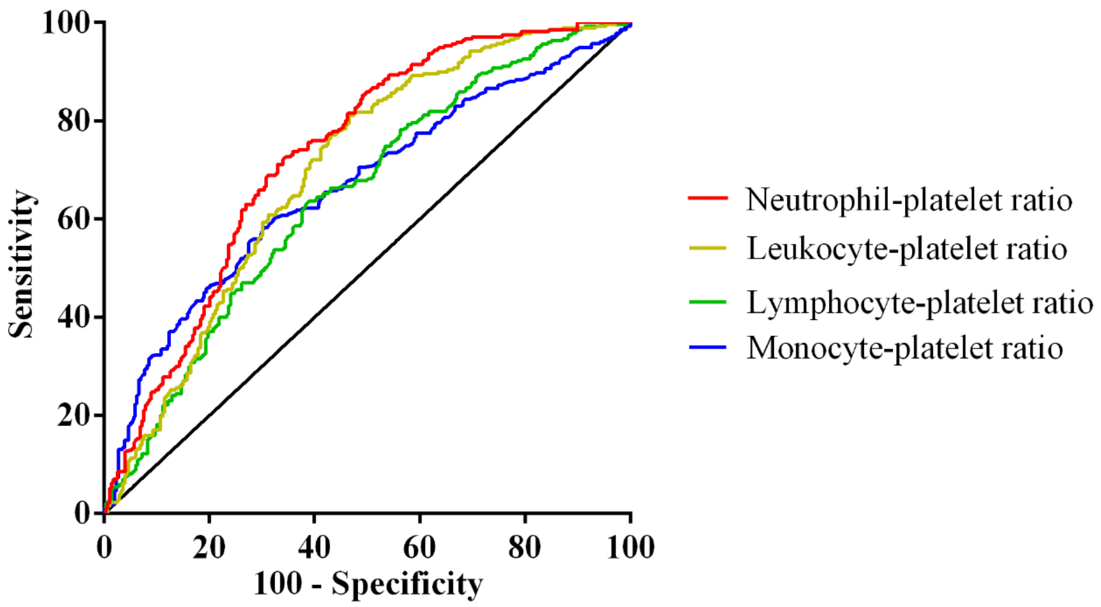

Supplementary Figure 2 Receiver operator characteristic analysis of blood test ratios for prediction of hemorrhagic transformation.
